# Alginate Spheres: Influence of Agar and Xanthan Gum Incorporation on Membrane Stability and Permeability

**DOI:** 10.3390/polym16192746

**Published:** 2024-09-28

**Authors:** Pascal Bevan, Idoia Codina-Torrella, Christina Xydia, Nisserine El Hammadi, María Pilar Almajano

**Affiliations:** 1 Chemical Engineering Department, Universitat Politècnica de Catalunya, Av. Diagonal 647, 08028 Barcelona, Spain; pascal.bevan@upc.edu (P.B.); christinexds@gmail.com (C.X.); elhammadi.nisserine@gmail.com (N.E.H.); 2 Agri-Food Engineering and Biotechnology Department, Universitat Politècnica de Catalunya, Esteve Terrades 8, 08860 Castelldefels, Spain

**Keywords:** spherification, calcium alginate, xanthan gum, agar-agar, delivery system, oolong tea

## Abstract

Calcium alginate spheres with a volume of about 5 mL can be used for important purposes. Those that incorporate oolong tea give, in addition to the recreational aspect, the possibility of drinking small quantities of this tea, because oolong tea can be used as a compound with antioxidant properties. This incorporation can be achieved by reverse spherification (5 mL). Six types of spheres have been made, all of them with calcium alginate and the presence or absence of agar-agar and xanthan gum—XG—in two concentrations. The weight loss of the spheres, the release of bioactive compounds over time (a total of 48 h), the surface (internal and external) of the membranes, and the physical characteristics of these membranes have been analyzed. The data obtained indicate that the presence of XG prevents the formation of precipitates inside the spheres and slows down weight loss. It also provides opacity to the spheres. However, the incorporation of agar-agar does not have a significant influence on the different parameters analyzed. The release of catechins reaches a maximum of 80% of what could be achieved under ideal conditions, and it reaches 90% in the first 3 h. The incorporation of XG increases the shelf life of the spheres, slows down the release of chelate, and decreases weight loss over time, allowing for a new perspective on the spherification process.

## 1. Introduction

Calcium alginate spheres, containing about 5 mL of an internal liquid, allow an explosion of flavor in the mouth, and it is important to note that they are increasingly used at social events. On the other hand, more and more people are becoming aware of the importance of natural antioxidants in their diet. For this reason, interest in natural sources with high antioxidant power has increased in several sectors to replace synthetic antioxidants, even in industrial applications. Oolong tea infusion has polyphenols, which consist of a mixture of catechins, and it is widely used for its antioxidant properties and absence of toxic side effects [1]. Studies have shown the numerous benefits of oolong tea, such as antioxidant, bacteriostatic, and anti-tumor activities and regulation of lipid metabolism [2]. For the development of novel functional products, there is currently a lot of interest in the inclusion of plant extracts with high antioxidant capacities. The incorporation of these extracts makes their protection against environmental and processing conditions necessary to preserve their stable properties over time. Different techniques have been proposed, of which encapsulation is one of the most widely used, especially in food applications [3]. Ionic gelation methods can be used to ensure the protection of bioactive substances, and to control the kinetics and release of these ingredients to the food matrix. Hydrogels with particular structural and mechanical characteristics could be used to safeguard the bioactive components that can be included within them [4]. In the literature, several authors have studied the encapsulation of plant extracts and monitored the diffusivity of these bioactive compounds [5,6,7,8,9,10]. Nutrizio et al. (2023) microencapsulated rosemary extracts by the ionic gelation method and investigated the release of polyphenols in simulated gastrointestinal tract conditions. The results of this study indicated that the incorporation of aqueous rosemary extracts in microcapsules is efficient and functional in terms of bioactive content and release properties [5]. Wu et al. (2023) successfully encapsulated naringenin, a flavonoid compound extracted from fruits, in zein–sodium alginate nanoparticles. The encapsulation of this natural antioxidant compound in nanoparticles enhanced its antioxidant capacity and achieved its sustained release, enabling the industry to successfully integrate naringenin into functional foods [6].

Spherification is a widely used encapsulation method in which a polymer hydrogel film covers a liquid, creating a sphere with a liquid core [11,12]. Alginate is a linear anionic polymer, a natural polysaccharide derived from brown algae, which is widely used for this purpose due to its biocompatibility, low toxicity, relatively low cost, and mild gelation by the addition of divalent cations [13]. Alginates consist of 1,4-β-D-mannuronic acid (M) and 1,4-α-L-guluronic acid (G) monomers, with a homogeneous (GG, MM) or heterogeneous (MG) block composition [13] in a random order. Gelation occurs when G-blocks coordinate with calcium ions, forming calcium alginate, a three-dimensional gel network known as an egg-box structure [11]. Moreover, it is worth noting that more attention is being paid to preparing films (not spheres) with two or more polymers in order to improve the physical and mechanical properties of alginate-based hydrogels [1].

On the other hand, it should be noted that the process of spherification with alginate, with spheres of a volume of more than 4 mL, has been successfully carried out for some years now. The incorporation of an alcoholic tea infusion inside these spheres with more than one polymer on their surface is a novelty in what has been achieved so far. The main objective of this incorporation is to keep the spheres’ physical characteristics unaltered as long as possible so that they can be stored for later use in social events. Moreover, the spherical structure of the alginate hydrogel membrane acts as a protective barrier, shielding the liquid core from environmental degradation caused by oxygen, light, or moisture, which can compromise the effectiveness of active ingredients in other encapsulation systems [14].

The flow of liquid loss from these spheres follows Poiseuille’s law, where the rate of liquid flow depends on the pore size and membrane thickness, which can be controlled by adjusting the concentration of polymers like alginate and xanthan gum. Xanthan gum, in particular, enhances the viscosity of the liquid core, further improving the retention of the bioactive compounds within the spheres. In this case, the flow of liquid volume loss approximates Poiseuille’s law (1):

Q = K·(R^4^·S)/η·∆P/e (1)

where Q is the flow of liquid that passes through the coating through porosity; S corresponds to the usable coating surface in m^2^; η corresponds to the dynamic viscosity of the fluid to be spherified; e is the thickness of the coating; R corresponds to the mean radius of the coating pore; ∆P corresponds to the pressure difference between the inside of the pearl and the outside; and K is a constant for every liquid.

It is important to take into account that xanthan gum (XG) is a thickener; the greater the XG concentration, the greater the viscosity of the liquid.

In this study, we aim to encapsulate tea extracts in edible polymer-based spheres using the reverse spherification method, prepared from different combinations of calcium alginate, agar, and xanthan gum (XG). By evaluating the physical stability of the spheres and monitoring the release of bioactive radical-scavenging compounds over time, we seek to explore the potential of this novel encapsulation system to improve retention, increase the time the sphere remains as such, and, also, explore its potential as a system for the controlled release of catechins in food matrices.

## 2. Materials and Methods

### 2.1. Materials

Sodium alginate “Brenntag 6021” was purchased from Brenntag (Paris, France). It has a viscosity between 150 and 300 mPa·s and a pH between 6 and 8.5 (in 1% solution, m/V). Its average molecular mass is 800 kDa. Vinpai Cimalgin, purchased from Vinpai (Saint-Dolay, France), has a viscosity between 150 and 350 mPa·s and a pH between 6 and 8 (in 1% solution, m/V). Its average molecular mass is 950 kDa. Xanthan gum was purchased from Corquimia Industrial S.L. (Barcelona, Spain). It has a viscosity between 1200 and 1700 mPa·s (in 1% solution with 1% KCl), with an average molecular weight of 240 kDa. Agar-agar was purchased from Ingredíssimo (a Brand by Foodíssimo, Barcelona, Spain), with an average molecular mass of 176 kDa. All other chemicals used were of analytical quality and purchased from Sigma-Aldrich (Barcelona, Spain).

### 2.2. Preparation of Spheres and Encapsulation of Tea Extracts

Calcium alginate liquid-core hydrogel spheres were formulated, using the frozen reverse spherification method, with calcium lactate serving as the cross-linking agent. In this study, six types of calcium alginate spheres were developed: (1) calcium alginate spheres with agar, (2) with agar and 0.07% XG, (3) with agar and 0.14% XG, (4) regular calcium alginate spheres, (5) with 0.07% XG, and (6) with 0.14% XG. The preparation process involved creating two solutions: the internal liquid for the spheres and the alginate bath. The alginate bath was prepared by dissolving 0.3% *w*/*v* of “Brenntag 6021” and 0.3% *m*/*v* of “Vinpai Cimalgin” sodium alginate powder in distilled water using an electric hand stirrer, resulting in a 0.6% *w*/*v* alginate solution. The internal liquid of the spheres was prepared by the incorporation of a tea infusion. To obtain the tea extracts, 0.5% *w*/*v* of raw powdered oolong green tea was infused in boiling water, using a continuous magnetic stirrer for 10 min. Afterward, the tea infusion was centrifuged (Orto Alresa Mod. Consul, Ajalvir, Madrid, Spain) at 2500 rpm for 5 min to remove any solid tea residues.

For the internal liquid, calcium lactate (0.6% *w*/*v*) was dissolved in the mixture comprising 65.2% tea infusion, 18.2% ethanol (*w*/*w*), and 16% *w*/*v* sugar syrup with a continuous magnetic stirrer. After the mixture was prepared, 5 mL of this mixture was poured into hemispherical silicone molds and frozen at −30 °C. These frozen hemispheres were then introduced into the alginate bath and maintained at approximately 30 °C, where they transformed into spherical shapes due to the surface tension gradient. The spheres were gently stirred in the alginate bath for 5 min, during which time the calcium ions in the mixture reacted with the alginate to form a membrane or shell around the liquid core. This process yielded pearls larger than 2 cm in diameter, with a liquid center encased by a calcium alginate membrane. After removing the spheres from the alginate bath, they were rinsed in a bath of distilled water and stored in jars filled with distilled water at room temperature

For tensile testing, elongated polymer films were required. These films were obtained by following the same preparation process as described for the spheres, with the only difference being that the mixture was poured into rectangular silicone molds (4 × 2 × 2 cm^3^), each filled with 15 mL of the prepared internal liquid.

For spheres incorporating agar and/or XG, the specified amounts of these compounds were added to the alginate bath to create a copolymer.

The preparation of agar/alginate composite beads included an additional step: a solution of agar (2.4% *w*/*v*) was prepared by heating distilled water to 50 °C for 15 min while slowly adding agar with continuous stirring. Once dissolved, the warm agar solution was introduced to the alginate bath at 30 °C, resulting in a final agar concentration of 0.2% *w*/*v*. After removal from the agar/alginate bath, the spheres were rinsed in distilled water at 15 °C to ensure complete agar solidification.

The manufacturing process of XG/alginate composite beads follows the same process as the alginate spheres with the addition of XG powder to the internal liquid. The XG was dissolved using an electric hand stirrer, and the resulting mixture was placed in a vacuum camera for complete de-aeration. The mixture was then transferred into hemispherical silicone molds and frozen at −30 °C, completing the preparation process.

### 2.3. Physical Characterization of Spheres

The weight loss and physical appearance of each type of sphere were systematically analyzed. To evaluate the permeability of the calcium alginate hydrogel membranes for the internal liquid, a weight loss experiment was conducted. This study aimed to quantify the diffusion rate of water molecules across the hydrogel membranes, providing an estimate of the water barrier properties of each type of sphere. For this purpose, four spheres from each composite group were weighed using precision scales under controlled laboratory conditions (23–25 °C). The weight of the spheres was recorded at five-minute intervals over a three-hour period, and weight loss was measured in grams. The visual morphology and color of the spheres were also monitored through direct observation over a two-month period to assess any changes in physical appearance over time.

### 2.4. Mechanical Properties of Spheres

The elongation at break (εB) and maximum tensile strength (σM) of the spheres were determined using a Zwick Roell uniaxial tensile machine equipped with test Xpert testing 1.2. software. The sensitivity parameters were set as follows: final test phase yield stress at 50%, force threshold for breaking detection at 0.01%, test speed at 50 mm/min, and preload at 0.05 MPa. To perform the tensile test, longer spheres were specifically used. The polymer films were cut into rectangular strips 50 × 15 mm^2^ in size for stress–strain measurement, following the method described by Bt Ibrahim et al. [14]. These strips were placed between two clamps aligned along the axis and held securely at both ends. The tensile stress and strain at break were calculated using the slope of the linear portion of the stress–strain curve. Fracturability testing of the spheres was carried out using a texturometer designed specifically for this purpose. The breaking force of ten samples from each composite bead type was measured to assess fracturability.

### 2.5. Diffusivity Assay

A diffusivity assay was conducted to evaluate the release of antioxidant bioactive components from oolong tea spheres. Six types of composite spheres containing tea extract were tested using distilled water as the simulant, in accordance with the European Commission Regulation (EU) No. 10/2011 on plastic materials and articles intended for contact with food. The spheres were submerged in water for a period of 48 h, and samples were collected at regular intervals: every 10 min during the first hour, every hour up to 3 h, and subsequently every 24 h. The collected samples were stored at −80 °C until further analysis. All experiments were performed in triplicate.

#### 2.5.1. Content of Total Phenolic Compounds

Total phenolic compounds (TPCs) were quantified by colorimetric spectrophotometry following the Folin–Ciocalteu method [9]. Water with the diffusion samples (7.6%, *v*/*v*) was mixed with a Folin–Ciocalteu-reactive (30.8, *v*/*v*) sodium carbonate solution at 20% (30.8% *v*/*v*) and ultrapure water (30.8%, *v*/*v*). Mixtures were allowed to stand for 1 h at room temperature and protected from light. The absorbance was measured in quadruplicate at 765 nm, at room temperature, using a spectrophotometer (FLUOstar OMEGA, Perkin-Elmer, París, France). TPC was expressed as mg of Gallic Acid Equivalent (GAE) per mL of the sample. The standard curve was obtained by plotting the absorbance against different concentrations of gallic acid ranging from 0.0025 to 0.025 M (R^2^ = 0.9952).

#### 2.5.2. Characterization of Phenolic Compounds by HPLC

HPLC was used to identify the different catechins found in the spheres’ external liquid. The identification and quantification were carried out using Water Alliance 2695 Series HPLC-MS equipment that consisted of an automatic sample injection system. The components were separated by a Water Symmetry C18 column (150 mm × 3.95 mm). HPLC conditions were set as follows. The mobile phase was composed of phase A (ultrapure water acidified with 0.1% formic acid) and phase B (acetonitrile acidified with 0.1% formic acid). The elution gradient corresponded to 95% A and 5% B, and the flow rate was 0.8 mL/min. An injection volume of 25 µL was added into the analytical C18 column at room temperature. Different commercial standards were subsequently used to identify and quantify the compounds (including EGC: (−)epigallocatechin; EGCG: (−)epigallocatechin gallate; CAF: caffeine; EC: (−)epicatechin; and ECG: (−)epicatechin gallate). The identification of the components was confirmed by matching their retention time (RT).

### 2.6. Scanning Electron Microscopy (SEM)

SEM was used to capture images of the surface and cross-section of films (JSM-7100F, JEOL, Tokyo, Japan). Scanning electron microscopy analysis methods are able to observe the changes in the surfaces of the pearls that take place because of the application of different treatments. Three scanning electron microscopy pictures of each kind of calcium alginate composite film were taken. The samples of the composite films were freeze-dried and placed on a double-sided adhesive tape fixed in the aluminum SEM holders and introduced into an evaporator to obtain a graphite coating. The voltage and the magnification used were 15 kV and 2500×, respectively.

### 2.7. Differential Scanning Calorimetry (DSC)

The thermal properties of the films were analyzed using Differential Scanning Calorimetry (DSC), following the methodology described by Segovia [15]. A Mettler–Toledo DSC30 Thermal Analysis System (Schwerzenbach, Switzerland) was employed for this purpose. Lyophilized films (10.00 ± 0.25 mg) were placed in 40-microliter open aluminum DSC crucibles. All samples were treated under a nitrogen atmosphere with a flow rate of 50 mL/min. The measurements were conducted over a temperature range of 30–300 °C, with a heating rate of 10 °C/min. Fusion enthalpies were calculated by integrating the calorimetric signal using a straight baseline, processed with STARe 3.15 software (Mettler Toledo, Barcelona, Spain).

### 2.8. Statistical Analysis

Statistical analyses were conducted by using Minitab statistical software, version 18 (Minitab Inc., Sate College, PA, USA). The data were reported as means ± standard deviation. The results were subjected to analysis of variance (ANOVA), and then a post hoc Tukey’s test was applied to determine significant differences among formulations (*p* < 0.05). 

## 3. Results and Discussion

### 3.1. Characterization of the Spheres

#### 3.1.1. Mechanical Properties

The effect of agar and XG content on the mechanical behavior of calcium alginate-based films was investigated in terms of the Young or elastic modulus (Et), the elongation at break (εB), and the maximum tensile or breaking strength (σM) values. Tensile strength represents the resistance to tension forces, Et represents films’ resistance to being elastically deformed, and εB% is related to films’ stretching ability, which represents the capacity of a film to be extended before breaking [16].

Table 1 shows Young’s modulus (Et), maximum tensile or breaking strength (σM), and elongation at break (εB) values of the samples. The Young’s modulus values suggested that the incorporation of XG increased the elasticity of the calcium alginate films in the spheres. The results indicate that the higher the XG concentration, the higher the Young’s modulus values. On the contrary, increasing the XG concentration in the spheres decreased their elongation at break. Values of tensile strength ranged from 0.120 to 0.243 MPa, with the lowest value for regular calcium alginate film and the highest for the 0.14% XG film. The combination of sodium alginate and XG increased tensile strength and Young’s modulus values, probably due to the compatibility and chemical synergetic interactions between the components [16]. In the same way, the studies conducted by Shan et al. showed that regular alginate film (without XG nor agar) presented lower tensile strength and Young’s modulus values, but higher elongation at break [17]. Although the grouping information obtained from the statistical analysis did not show any significant difference between the Young’s modulus values of the different composite films, the increase in these values when increasing the concentration of XG can be remarked. Liu et al. showed a significant increase in tensile strength and elongation at break for composite films combining sodium alginate with graphene oxide, and alginate with ammonia-functionalized graphene oxide [18]. This may be due to the occurrence of synergistic effects due to interactions between the different chemical compounds. Similar results were reported by Galus et al., who combined pectin with alginate to create composite films with higher tensile strength and elongation at break [16].

It can be observed that the addition of XG and/or agar during the gelification process of calcium alginate increased the hardness of the hydrogel, making it stronger but more breakable.

Table 2 shows the data obtained in the fracturability test of the spheres, which was carried out by measuring their breaking force (σM).

These results show that the incorporation of XG in the internal liquid of the spheres leads to an increase in their pressure resistance, providing greater mechanical properties. More specifically, the higher the XG concentration, the higher the force necessary to break the spheres. On the contrary, the addition of agar in the manufacturing process seems not to have led to a reduction in the mechanical properties of the spheres, as the required breaking force values are not significantly different.

In the current study, the breaking force between composite films with and without agar content was significantly different. Values of the breaking force ranged significantly (*p* < 0.05) from an average of 581.6 to an average of 334.3 kPa, with the lowest value for the spheres with agar and the highest for the spheres without it. The breaking force of the beads without agar was significantly higher, meaning that they showed greater mechanical strength.

The influence of XG on the breaking strength of the spheres is less pronounced compared to the effect of agar presence or absence. Significant differences in breaking strength are observed only in the sample with the highest concentration of XG and no agar. The other two samples, which also lacked agar (one without XG and one with 0.7% XG), did not exhibit significant differences in breaking strength.

#### 3.1.2. Weight Loss at Atmospheric Pressure

**Figure 1:**
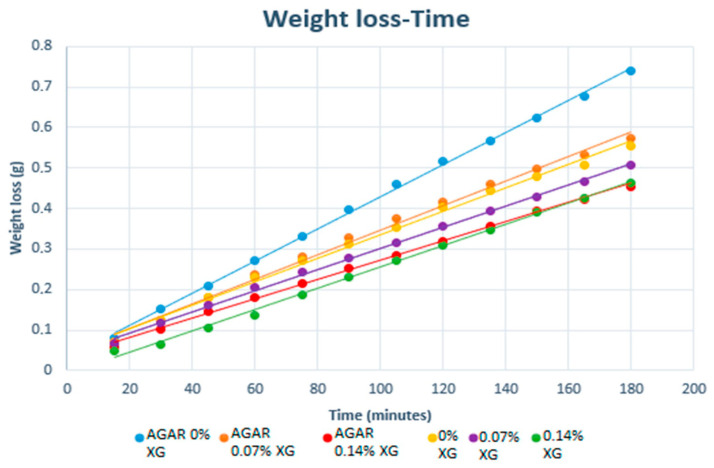
Weight loss of each composite bead over 3 h of being exposed to atmospheric pressure with *n* = 4.

Figure 1 shows the average weight loss of each type of sphere vs. time (min).

Figure 1 shows that the weight loss of each kind of composite bead increased linearly. In all of them, a decrease in weight can be observed up to 3 h. It is observed that the film compositions in which weight loss takes place with the lowest speed in this period of time are those with a higher XG concentration since they present a lower slope than the others. Again, as in the previous section, the presence or absence of agar has a relatively low influence compared to the presence of XG.

It was noted that the addition of agar was not as influential as the addition of XG. Therefore, the slopes of the lines obtained were statistically analyzed and classified according to the concentration of XG. The results are shown in Table 3. The grouping information obtained indicated that the weight loss rate between composite films with 0% XG and 0.14% XG is significantly different. Overall, these results indicate that the increase in the XG concentration of the alginate composite films is inversely proportional to their weight loss, and this is indicative of the fact that the water permeability (which is the component that passes through the membrane) is influenced by the amount of XG.

The fact that the spheres containing a higher amount of XG show less weight loss may be due to the following:

- XG reportedly has a good water-binding capacity [19,20].
- XG increases the viscosity of liquid according to Poiseuille’s law.

A corresponding statistical analysis was performed on the slope of the weight loss curve vs. the agar content of each sample. The grouping information did not show any significant difference between the weight loss rate between the films that contain agar and those that do not, as their average slope values shared the same letter. However, it can be remarked that the agar composite films have a higher weight loss rate per time.

### 3.2. Diffusivity Assay Results

The study of diffusivity was conducted in distilled water. The analysis of the diffusivity was carried out every 10 min the first hour and later every hour until 3 h, taking a sample at 24 h and 48 h. The analysis conducted was the qualitative analysis of TPC.

In all cases, a similar behavior is observed, characterized by a rapid diffusivity in the first hour, with a decrease in the rate of passage of bioactive compounds through the membrane in the following hours, until pseudo-stabilization is reached after 3 h. Samples taken after 24 and 48 h show that the increase achieved is much lower. In order to compare these speeds, two intervals were used: the first 50 min and from then until 3 h, when, in addition to the qualitative analysis of the compounds with antiradical activity (by TPC), the concentration of the catechins was also determined by HPLC-DAD.

**Figure 2:**
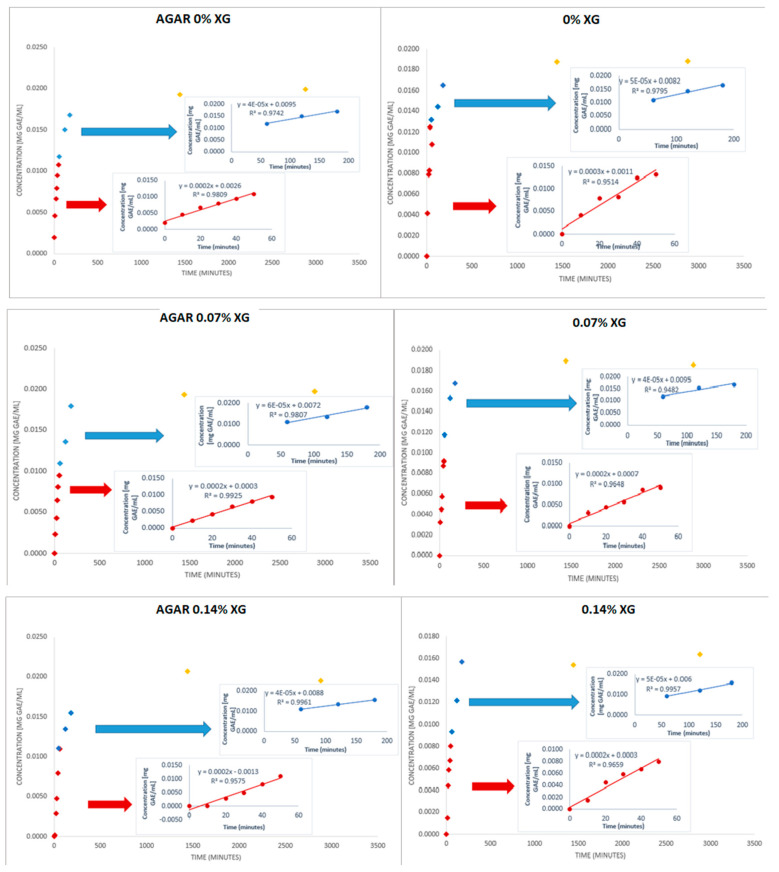
Evolution of total phenolic compounds (TPCs) of the different treatments over a storage time of 48 h with *n* = 2. If “agar” appears, it means that the samples have been made in an agar + alginate bath. The percentages are 0% XG, 0.07% XG, and 0.14% XG. The TPC is represented as concentration of gallic acid (mg GAE/mL) over time.

Table 4 shows a summary of the slopes of the straight lines in the different sections and different treatments. Figure 2 shows an overall representation of the diffusivity behavior in water.

- Section 1: from 0 to 50 min, marked in red.
- Section 2: from 1 to 3 h, marked in blue.
- Section 3: from 24 to 48 h, marked in yellow.

Figure 2 shows the release behavior of TPC in these spheres. In all of them, the highest velocity of the diffusivity phenomena can be observed up to 3 h. Thereafter, there is no longer any significant increase. In Section 2 (50 min to 3 h), the diffusivity decreases by approximately 70–80% compared to Section 1 (first 50 min). As observed, the maximum release of the polyphenolic compounds occurred during the first 3 h. From that moment, this content remained similar in all samples.

Similar results were obtained by other authors, who reported that at the beginning of the phenolic content release in a food simulation solution, there was a sudden rapid release phenomenon, followed by a sustained slow release, which finally reached an equilibrium state [21,22].

The data in Table 4 indicate that there are no significant differences in diffusivity across the different types of films. In the first section, up to 50 min, if we look at the slope of the straight line, it is observed that the diffusion rate is so great that no differences between the different treatments of the spheres can be detected, as the sensitivity provided is not sufficient.

A trend is observed: it seems that the diffusivity is higher (at end time) in the agar samples, while the presence of XG slightly hinders the outflow of bioactive compounds into the water.

In 2022, Kolar et al. found similar results working with Stevia rebaudiana extracts encapsulated in microspheres whose shell was calcium alginate with and without XG. In particular, they reported that the microsphere shell structure and surface morphology are altered by the addition of XG, which slows down the release of TPC [23].

It is striking to observe that the addition of agar-agar in the process of calcium alginate gelification does not reduce the diffusivity of the hydrogel, meaning that agar-agar addition to the hydrogel is not useful if our purpose is to have slow diffusion over a long time.

Table 5 shows the concentration of catechins after keeping the spheres in contact with distilled water for 3 h. Given that the procedure used is to add 3 times the weight of the sphere in distilled water, it can be considered that the total volume is 4 times the initial volume, which implies that the initial concentration would be distributed in a volume 4 times greater and, therefore, if the distribution were perfectly homogeneous, the initial concentration of the liquid inside the sphere would decrease to a quarter, considering the total volume. Taking into account this criterion, the maximum cannot be reached in any of the cases. It is between 20% lower than the total equilibrium (catechins homogeneously distributed throughout the volume) and 50% lower. Two potential explanations account for this discrepancy. First, a portion of the catechins may remain within the alginate shell, which was not quantified. Second, the formation of catechin polymers, particularly in the absence of xanthan gum (XG), may lead to precipitates that are unable to permeate the alginate membrane, whether or not agar is present. No significant differences were found between the samples, although a trend was observed. The presence of agar facilitates the passage through the membrane, while XG facilitates the retention of catechins.

### 3.3. Surface Morphology

**Figure 3:**
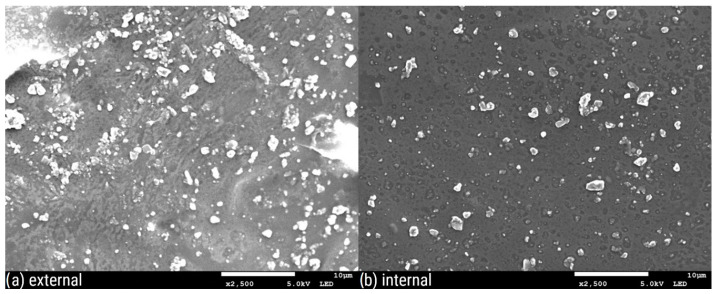
SEM pictures of calcium alginate composite film with agar and without XG: (**a**) external and (**b**) internal side of the film.

**Figure 4:**
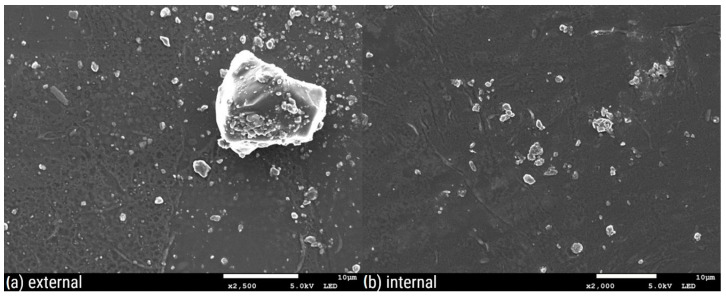
SEM pictures of calcium alginate composite film with agar and with 0.07% XG: (**a**) external and (**b**) internal side of the film.

**Figure 5:**
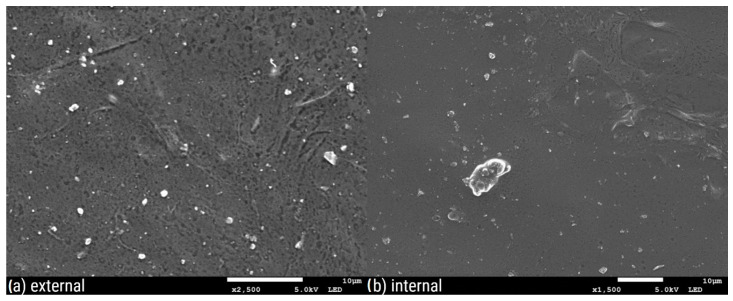
SEM pictures of calcium alginate composite film with agar and with 0.14% XG: (**a**) external and (**b**) internal side of the film.

**Figure 6:**
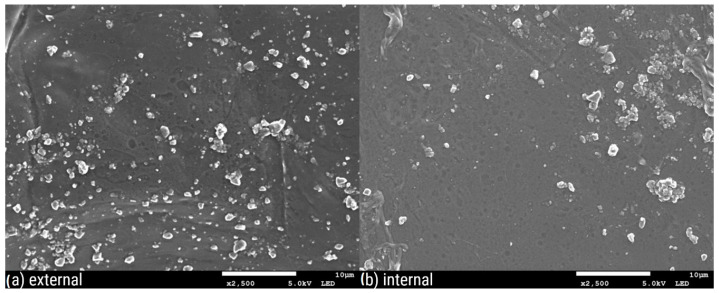
SEM pictures of calcium alginate composite film: (**a**) external and (**b**) internal side of the film.

**Figure 7:**
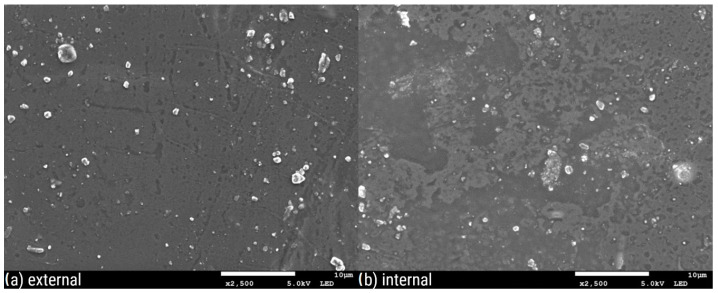
SEM pictures of calcium alginate composite film with 0.07% XG: (**a**) external and (**b**) internal side of the film.

**Figure 8:**
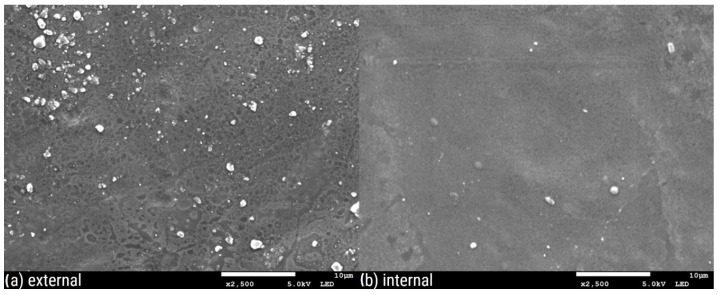
SEM pictures of calcium alginate composite film with 0.14% XG: (**a**) external and (**b**) internal side of the film.

Figure 3, Figure 4, Figure 5, Figure 6, Figure 7 and Figure 8 show three SEM images for each kind of composite calcium alginate film at 7 days of storage.

Most of the samples present some wrinkles on the surface, which were probably caused by the placement of the membranes on the SEM holders, due to their thinness and fragility. It is remarkable that small molecules are observed on the surface of all the composite films, the frequency of which varies between the samples. These molecules probably come from the deposition of calcium lactate in both the external and internal sides of the spheres over time. Another possible explanation is that these compounds come from the precipitation of the tea over time. Most molecules are detected in the samples with 0% XG. As the concentration of the specific component increases in the film composition, the presence of these molecules on their surface seems to decrease. Also, it should be noted that the presence of these molecules is more frequent in the samples obtained from the external side of the film. This observation may be attributed to the external surface of the bead being in contact with the liquid, which lacked XG. No significant differences were observed between the surfaces of films containing agar and those without.

### 3.4. Appearance of Spheres

**Figure 9:**
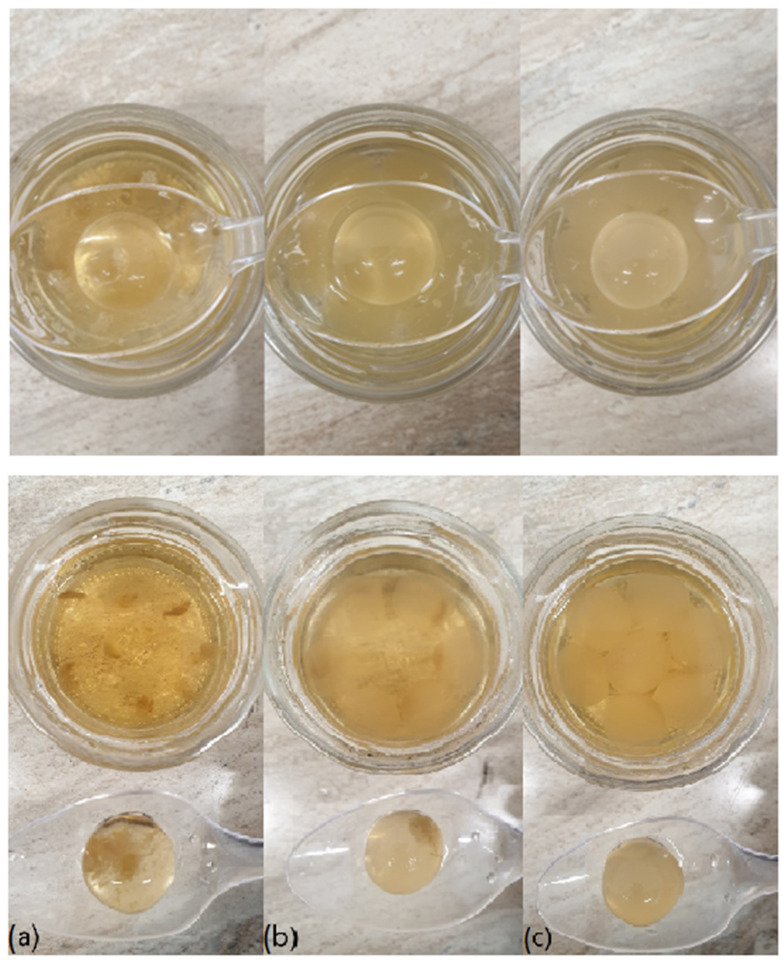
Spheres immediately after their production (first row) and 1 week after their production in distilled water (second row). (**a**) Without XG, (**b**) 0.07% XG, (**c**) 0.14% XG.

The spheres were stored in glass jars at 23–25 °C (laboratory conditions) for 1 week in distilled water. Figure 9 shows the appearance of the spheres with different XG concentrations immediately after their production and 1 week later. It can be observed that the addition of XG in the internal liquid imparts a less transparent appearance to the spheres: the higher the concentration of XG, the less transparent the spheres.

The photos of the spheres incorporating agar have no difference in appearance with non-agar- spheres.

The second row of Figure 9 shows the spheres after one week of storage in distilled water at room temperature. It should be noted that in the absence of XG, a precipitate appears, which may be due to a conglomeration of the catechins present in the tea infusion. This precipitate decreases with increasing XG concentration. In the samples with 0.14% XG, practically no clumping is observed.

The formation of the precipitate is presumably due to a reduction in the solubility of the tea and the polymerization of catechins. XG appears to stabilize these substances, keeping them globally dispersed in the liquid and preventing the formation of a visible precipitate. At 0.07% XG, partial stabilization is achieved, which becomes complete at 0.14% XG, thereby confirming the effectiveness of XG as a stabilizing agent.

It is noteworthy that the presence or absence of agar does not significantly affect the appearance of the spheres.

### 3.5. Thermal Properties of the Films

DSC of the coating of the spheres of the six treatments after a freeze-drying process was carried out. In order to compare with the starting products and to see possible relationships, DSC of the initial products, both alginates, agar, xanthan gum, and calcium lactate, was also carried out. The study of the peaks does not allow us to determine the existence of any new specific bond resulting from the incorporation of agar or XG, as the only peaks detected correspond to the dehydration of both alginates followed by another one corresponding to the degradation of the same. But it could be said that the addition of agar and XG could improve the thermal stability of films. The effect on thermal properties depends on the internal structure. It is possible that agar and XG provide higher stability and require more energy to break the structure, resulting in higher thermal stability.

Both sodium alginate spheres have a low level of humidity, lower than 9%. In the case of reverse spherification where there is a sodium alginate bath in order to have an important source of alginate ion, the humidity is not an issue. Nevertheless, the humidity of calcium lactate is around 15%, and in the case of reverse spherification, where calcium ions are in a limited quantity to control the coating quality and thickness of the spheres, it is important to take it into account. Up to 200 °C, both sodium alginate spheres start the degradation process as combustion with a peak value around 252 °C.

A degradation process for calcium lactate was not observed in the temperature range up to 300 °C but a fusion process occurred around 135 °C.

## 4. Conclusions

This study represents the first documented use of agar-agar and xanthan gum (XG) in the reverse spherification process, to the best of our knowledge. The resulting spheres, approximately 5 mL in volume, can serve as pseudo-slow-release agents for bioactive compounds. The presence or absence of agar-agar and XG does not significantly impact the release kinetics. Within the first hour, more than 60% of the total release observed over 48 h is achieved, and by the end of 3 h, approximately 90% release is observed for all types of spheres.

The trends identified include the following: (1) The presence of agar-agar facilitates the passage of polyphenols through the membrane, suggesting that a network with fewer cavities is not formed as expected. (2) The presence of XG prevents the formation of catechin polymers (precipitates) inside the sphere, although it introduces a degree of opacity. Despite this, catechins that pass through the membrane do not reach the theoretical maximum, achieving up to 80% of the potential release.

Electron microscopy analysis reveals that the inner surface of the film is more homogeneous and contains fewer particles compared to the outer surface. Spheres exposed to atmospheric pressure lose weight due to liquid passage through the membrane, with XG significantly reducing this weight loss.

The results indicate that XG enhances the durability of spheres containing oolong tea extract, both in air and while stored in water. In contrast, while agar-agar complicates the manufacturing process, it does not significantly affect the outcomes of the analyses performed.

It has been shown that the presence of XG can facilitate the durability of spheres that incorporate alcoholic extract of oolong tea, both in air and stored in water, while the presence of agar-agar makes the manufacturing process more difficult, but it does not have a significant influence on the analyses carried out.

## Figures and Tables

**Table 1:** Young’s modulus (Et), maximum tensile or breaking strength (σM), and elongation at break (εB) values of the samples.

Sample ^1^	E_t_ (MPa)	σM (MPa)	εB (%)
Agar 0%	0.266 ± 0.045 ^a^	0.191 ± 0.081 ^b^	107.7 ± 32.1 ^c^
Agar 0.07%	0.282 ± 0.072 ^a^	0.168 ± 0.038 ^b^	50.8 ± 16.4 ^a,b^
Agar 0.14%	0.339 ± 0.160 ^a^	0.160 ± 0.025 ^b^	38.3 ± 7.8 ^a^
0%	0.193 ± 0.033 ^a^	0.120 ± 0.036 ^a^	58.9 ± 23.7 ^b^
0.07%	0.325 ± 0.092 ^a^	0.223 ± 0.078 ^c^	45.3 ± 14.9 ^a^
0.14%	0.372 ± 0.065 ^a^	0.243 ± 0.025 ^c^	36.0 ± 8.9 ^a^

^1^ In the samples, the percentage indicates the concentration of xanthan gum. If “agar” appears, it means that the samples have been made in an agar + alginate bath. For each column, values sharing the same superscript indicate that there is no significant difference between samples with α = 0.05.

**Table 2:** Average breaking force of each kind of sphere.

Sample ^1^	σM (kPa)
Agar 0%	282.6± 42.6 ^a^
Agar 0.07%	379.4 ±64.7 ^a^
Agar 0.14%	345.4 ± 116.1 ^a^
0%	458.4 ± 51.5 ^b^
0.07%	539.9 ± 35.7 ^b^
0.14%	764.9 ± 99.8 ^c^

^1^ In the samples, the percentage values represent the concentration of xanthan gum used. Samples indicated with the term “agar” were prepared in an agar + alginate bath. Values sharing the same superscript indicate that there is no significant difference between samples with α = 0.05.

**Table 3:** Average weight loss slope between samples with different XG concentrations.

Xanthan Gum	Average Slope ± Sd ^1^
0%	3.43 × 10^−3^ ± 7.89 × 10^−4 a^
0.07%	2.81 × 10^−3^ ± 3.68 × 10^−4 a,b^
0.14%	2.45 × 10^−3^ ± 2.56 × 10^−4 b^

^1^ Values sharing the same superscript indicate that there is no significant difference between samples with α = 0.05.

**Table 4:** Slope and ordinate at the origin of the TPC in the water in contact with the spheres vs. time.

Sample ^1^	Slope ^2^ (0–50 min)	Ordinate (0–50 min)	Slope ^2^ (1–3 h)	Ordinate (1–3 h)	Maximum Value ^3^ (mg GAE/mL)
Agar 0%	2 × 10^−4^	2.6 × 10^−3^	4 × 10^−5^	9.5 × 10^−3^	0.0207 ^a^
Agar 0.07%	2 × 10^−4^	3 × 10^−4^	6 × 10^−5^	7.2 × 10^−3^	0.0197 ^a^
Agar 0.14%	2 × 10^−4^	1.3 × 10^−3^	4 × 10^−5^	8.8 × 10^−3^	0.0199 ^a^
0%	3 × 10^−4^	1.1 × 10^−3^	5 × 10^−5^	8.2 × 10^−3^	0.0188 ^a^
0.07%	2 × 10^−4^	7 × 10^−4^	4 × 10^−5^	9.5 × 10^−3^	0.0189 ^a^
0.14%	2 × 10^−4^	3 × 10^−4^	5 × 10^−5^	6.0 × 10^−3^	0.0164 ^a^

^1^ Percentage values represent the concentration of xanthan gum in the samples. The term “agar” denotes that the samples were prepared in an agar-alginate bath. ^2^ In mg GAE/(mL × h). ^3^ At the maximum value, all types of spheres share the same superscript because no significant differences have been detected at α = 0.05.

**Table 5:** Catechin concentration determined by HPLC-DAD initially (inside the sphere) and after 3 h of the spheres being in contact with distilled water.

Sample ^1^	EGC ^2^	EGCG ^2^	EC ^2^	ECG ^2^	CAF ^2^
Initial infusion concentration	6.14	15.14	3.34	5.53	10.49
Inside the sphere	3.99	9.84	2.17	3.59	6.82
Agar 0%	0.71	1.78	0.41	0.61	1.35
Agar 0.07%	0.68	1.72	0.38	0.61	1.23
Agar 0.14%	0.67	1.68	0.35	0.55	1.25
0%	0.53	1.58	0.32	0.51	1.12
0.07%	0.49	1.49	0.33	0.48	1.11
0.14%	0.48	1.42	0.27	0.45	0.98

^1^ The percentages represent the concentration of xanthan gum in the samples. Samples labeled with “agar” were prepared using an agar–alginate bath. ^2^ In micrograms/mL, EGC: (−)epigallocatechin; EGCG: (−)epigallocatechin gallate; EC: (−)epicatechin; ECG: (−)epicatechin gallate; and CAF: caffeine. Mean *n* = 2. The SD was <5% of the mean.

## Data Availability

The original contributions presented in the study are included in the article, further inquiries can be directed to the corresponding author.
